# Total phenolic contents, cytotoxic, free radicals, porcine pancreatic α-amylase, and lipase suppressant activities of *Artemisia dracunculus* plant from Palestine

**DOI:** 10.3389/fphar.2024.1351743

**Published:** 2024-03-07

**Authors:** Nidal Jaradat, Majdi Dwikat, Johnny Amer, Mustafa Ghanim, Mohammed Hawash, Fatima Hussein, Linda Issa, Salsabeel Ishtawe, Shahd Salah, Sara Nasser

**Affiliations:** ^1^ Department of Pharmacy, Faculty of Medicine and Health Sciences, An-Najah National University, Nablus, Palestine; ^2^ Department of Allied Sciences, Faculty of Medicine and Health Sciences, An-Najah National University, Nablus, Palestine; ^3^ Department of Biomedical Sciences, Faculty of Medicine and Health Sciences, An-Najah National University, Nablus, Palestine

**Keywords:** Artemisia dracunculus, total phenols, DPPH free radical inhibitory, antidiabetic, antiobesity, cytotoxicicity

## Abstract

Artemisia dracunculus: L. (*A. dracunculus*) is a popular vegetable and spice cultivated across many Middle Eastern countries. The herb’s aqueous extract has significant folkloric medicinal importance for treating various disorders. Hence, the present investigation aimed to investigate *A. dracunculus* hydrophilic extract phytochemical constituents and pleiotropic biological potentials, as no previous studies have investigated the antilipase and anti-α-amylase effects of the *A. dracunculus* plant. Total phenol content and phytochemical screening assays were performed utilizing standard analytical methods. While the α-amylase inhibition, free radical-scavenging, antilipase, and cytotoxic activities were determined using dinitrosalicylic acid (DNSA), DPPH, p-nitrophenyl butyrate (PNPB), and MTS assays, respectively. The standard phytochemical analysis of *A. dracunculus* aqueous extract shows that this extract contains only a phenolic group. The total phenol content was 0.146 ± 0.012 mg GAE/g of the plant dry extract. The *A. dracunculus* aqueous extract exhibited potent DPPH free radical inhibitory (IC_50_ dose of 10.71 ± 0.01 μg/mL) and anti-lipase activities (IC_50_ dose of 60.25 ± 0.33 μg/mL) compared with Trolox (IC_50_ = 5.7 ± 0.92 μg/mL) and Orlistat (IC_50_ = 12.3 ± 0.35 μg/mL), respectively. However, it showed a weak anti-α-amylase effect (IC_50_ value > 1,000 μg/mL) compared with Acarbose (IC_50_ = 28.18 ± 1.27 μg/mL). *A. dracunculus* has a cytotoxic effect against the HeLa cancer cell line compared with the chemotherapeutic agent Doxorubicin. The extract has the same percent of inhibition as Doxorubicin (99.9%) at 10 mg/mL. Overall, these results pointed out for the first time the importance of considering *A. dracunculus* effects as a favorite candidate for preventing and treating metabolic disorders. Also, our results confirm the findings of previous reports on the role of *A. dracunculus* in the management of cancer and disorders resulting from the accumulation of harmful free radicals. On the contrary, the current study concluded that the antidiabetic role of *A. dracunculus* could be minimal. Further in-depth investigations are urgently warranted to explore the importance of A. dracunculus in pharmaceutical production.

## 1 Introduction

Folkloric medicinal plants are commonly used to treat many illnesses worldwide, allowing scientists and pharmacists to extract many drugs from these plants and use them in various medical and pharmaceutical fields (Petran et al., 2020). Many countries have, therefore, returned to using plant extract in disease treatment in the twenty-first century. According to the evidence-based applications, this is due to its significant benefits (Jaradat, 2015; Holtmann et al., 2020).

**FIGURE 1:**
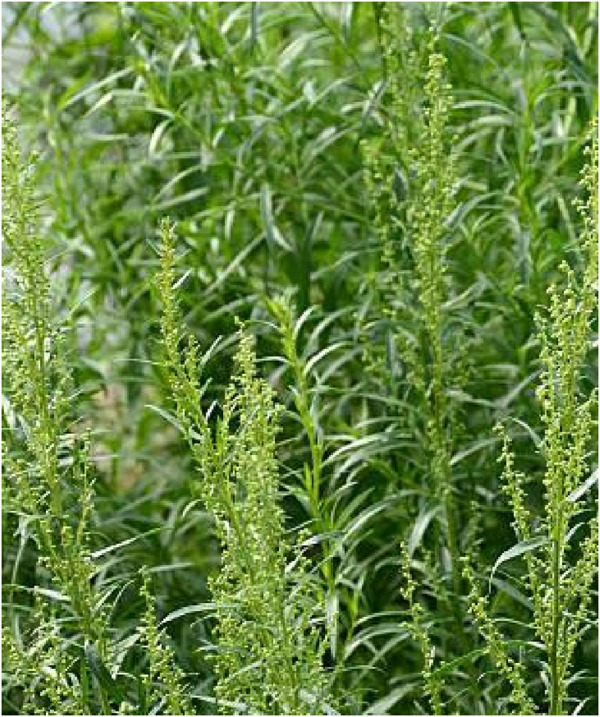
*A. dracunculus* aerial parts image.

*Artemisia dracunculus* L. (Tarragon) is a woody, perennial herb in the Asteraceae family that originated in North America and Eurasia. *A. dracunculus* varies in stem height between 40 and 150 cm. Aerial stems ascend from thick, horizontal rhizomes growing in clusters. The leaves are 1.2–8.0 cm long and 1–6 mm wide (Figure 1). In traditional medicine, *A. dracunculus* is used to treat digestive system disorders. Additionally, it was employed as a local anesthetic for alleviating toothaches, wounds, and cuts. The plant water extract is utilized in Arabic traditional medicine to treat insomnia, gingivitis, diabetes, and foot and mouth illnesses (Mohammed and Jamous, 2008; Ekiert et al., 2021).

The most important reported groups of biologically active secondary metabolites in *A. dracunculus* are essential oil, coumarins, flavonoids, phenolic acids, polyacetylene derivatives, flavonoids, vitamins, and tannins (Obolskiy et al., 2011).

Recent scientific studies have shown some significant properties of the *A. dracunculus* plant, including antibacterial, antifungal, antiprotozoal, antioxidant, immunomodulatory, and antineoplastic characteristics (Ekiert et al., 2021).

One of the most common problems that can widely spread these days is oxidative stress, which can affect normal cells and change them into cancerous cells because of the accumulation of a considerable amount of reactive oxygen species (ROS) (Kirtonia et al., 2020). These compounds are found in huge numbers due to the frequent anaerobic respiration, which helps the mitochondria provide energy to the tissue (Mailloux, 2020), and these radicals can be reduced by using plants with antioxidant effects. Thus, it may prevent the production of ROS and reduce its harmful effects (George and Abrahamse, 2020).

Another problem is obesity, an abnormal or increasing fat accumulation that may affect health status. It is described as a major risk factor for some chronic diseases like diabetes, cardiovascular diseases, musculoskeletal disorders, and cancer (Safaei et al., 2021). All over the world, obesity has approximately tripled since 1975. In 2016, more than 1.9 billion adults aged 18 and older were overweight. Above 650 million of these were obese, and over 340 million children and adolescents aged 5–19 were overweight or obese. Thirty-nine million children under 5 years old were overweight or obese in 2020 (World Health Organization, 2021c). According to the World Health Organization (WHO) assessment, one in every five adults will be obese in 2025 (Mohammed et al., 2018).

Diabetes mellitus (DM) also appears to be a big challenge for both poor regions and the richest countries. DM is a metabolic chronic disorder characterized by long-term hyperglycemia due to impaired insulin secretion and/or defects in insulin action. It disturbs the metabolism of fat, carbohydrate, and protein (Czech, 2017). The main complication of DM is a microvascular disease such as retinopathy, nephropathy, and neuropathy. Also, macro-vascular diseases like angina pectoris and myocardial infarction, transient ischemic attacks, strokes, and peripheral arterial disease (Wang et al., 2020). According to the WHO, the number of diabetics was 108 million in 1980 and 422 million in 2014. From 2000 to 2016, premature mortality from diabetes was a 5% increase. In addition, the number of deaths directly caused by diabetes in 2019 was 1.5 million (World Health Organization, 2021b). In addition, according to the CDC’s (Centers for Disease Control) National Diabetes Statistics Report for 2020, cases of diabetes were 34.2 million (Diabetes Research Institute, 2021).

Cancer is an abnormal growth of cells in the body. It is considered the second leading cause of death and is spreading globally, resulting in nearly 10 million deaths in 2020 and 300,000 cases being diagnosed among children. Nevertheless, 30%–50% of the cases can be treated and prevented (World Health Organization, 2021a).

The correlations between oxidative stress, obesity, diabetes, and cancer underscore the intricate interplay among these health conditions (Bao et al., 2011). Oxidative stress, resulting from an imbalance between the production of reactive oxygen species (ROS) and the body’s ability to neutralize them, is implicated in the pathophysiology of obesity, diabetes, and cancer (Crujeiras et al., 2013). In obesity, excess adipose tissue can contribute to oxidative stress by releasing pro-inflammatory cytokines and adipokines. This oxidative stress, in turn, may contribute to insulin resistance and the development of DM type 2 (Fernández-Sánchez et al., 2011). Moreover, persistent oxidative stress is recognized as a factor in the initiation and progression of cancer, as it can lead to DNA damage and mutations (Bartsch and Nair, 2006). Diabetes, with its associated hyperglycemia, further exacerbates oxidative stress, creating a conducive environment for cancer development (Giri et al., 2018). The intricate relationships among oxidative stress, obesity, diabetes, and cancer highlight the need for holistic approaches to health management and underscore the importance of lifestyle interventions and therapeutic strategies that target common pathways involved in these interconnected conditions.

Due to *A. dracunculus* folkloric medicinal and economic importance as a popular medical remedy, vegetable, and spice, the current study aims to investigate *A. dracunculus* hydrophilic extract phytochemical constituents, total phenols, antidiabetic (anti-α-amylase), anti-obesity (antilipase), DPPH free radical inhibitory (antioxidant) and cytotoxic activities.

## 2 Material and methods

### 2.1 Sample and extraction procedure

The *A. dracunculus* aerial parts (stems, leaves) were purchased from a herbal store in Ramallah/Palestine. This plant species was identified and confirmed by pharmacognosist Dr. Nidal Jaradat in the herbal products laboratory of the Department of Pharmacy at An-Najah National University and deposited within a voucher code of Pharm-PCT-2801. The dried plant parts were powdered using a mechanical blender. The extraction of the active substance in our study was based on using water. 400 g of dry *A. dracunculus* was weighed and steeped in 4,000 mL of boiled water for 12 h at room temperature (Nagai et al., 2005). The extract was filtered twice with filter paper and then dried in a dryer for 48 h. Finally, the extraction was kept in the refrigerator in a closed container at 4°C.

$$\%\text{ Yield}=\left(\text{Weight of }A.\;dracunculus\text{ extract }/\text{weight of dry plant}\right)\times 100\%$$

The *A. dracunculus* hydrophilic extract Yield was 11.325%.

### 2.2 Chemical and reagents

Benedict^’^s and Millon’s reagents were purchased from Gadot (United States). Ninhydrin solution, Molish^’^s reagent, H_2_SO_4_, chloroform, magnesium ribbon, HCl, FeCl_3,_ NaOH, and iodine solution were obtained from Alfa Aesar (England). Porcine pancreatic α-amylase enzyme, dimethyl sulfoxide (DMSO), Trolox ((s)-(−)-6 hydroxy-2,5,7,8-tetramethychroman-2-carboxylic acid), Folin-Ciocalteu’s reagent, porcine pancreatic lipase enzyme, dinitrosalicylic acid (DNSA), Doxorubicin, and 2, 2-Diphenyl-1-picrylhydrazyl (DPPH) were purchased from Sigma-Aldrich (Germany). Gallic acid, porcine pancreatic lipase enzyme, methanol, 1% L-glutamine, Dragendroff reagent, 1% Penicillin/Streptomycin, and sodium carbonate were brought from Merck (Darmstadt, Germany).

### 2.3 Phytochemical screening

The phytochemical determination of *A. dracunculus* hydrophilic extract was carried out using standard analytical methods to screen the presence of major phytochemical classes (Trease and Evans, 1983; Cartwright, 2016).

#### 2.3.1 Test for reducing sugars

1 mL of each of Fehling’s solutions, I and II, was added to 2 mL of the aqueous solution of the extract. The mixtures were heated in a boiling water bath for about 2–5 min. The production of a brick-red precipitate indicated the presence of reducing sugars.

#### 2.3.2 Alkaloids test

The 0.5 g of the plant extract was dissolved in 5 mL of 1% HCl and kept in a water bath for about 2 minutes. 1 mL of the filtrate was treated with Dragendroff’s reagent Turbidity or precipitation was taken as an indicator for the presence of alkaloids.

#### 2.3.3 Tannins and phenols test

About 0.5 g of the sample was dissolved in 10 mL of boiling water and was filtered. A few ml of 6% FeCl_3_ was added to the filtrate. The deep green color appeared to confirm the presence of Tannins.

#### 2.3.4 Flavonoids test

About 0.2 g of the extracts were dissolved in methanol and heated for some time. A chip of Mg metal was introduced, followed by adding a few drops of conc. HCl. The appearance of red or orange was an indicator of the flavonoids.

#### 2.3.5 Saponin test

About 0.5 g of the plant extract was stirred with water in the test tube. Frothing persists on warming was taken as a piece of evidence for the presence of saponin.

#### 2.3.6 Steroids test

The Salkowski technique was utilized for the identification of steroids. A solution containing 0.5 g of extract was dissolved in 3 mL chloroform and then filtered. Concentrated H_2_SO_4_ was added to the filtrate to create a distinct layer below. A good indication of the presence of a steroid ring was determined based on the observation of a reddish-brown tint.

#### 2.3.7 Test for glycoside

About 0.5 g of the extracts were dissolved in 2 mL of glacial acetic acid containing one drop of 1% FeCl_3_ and concentrated H_2_SO_4_. A brown ring obtained at the interphase indicates the presence of deoxy sugar. A violet ring appeared below the ring, while in the acetic acid layer, a greenish ring appeared just above the ring and gradually spread throughout this layer.

### 2.4 Determination of total phenol content

The amount of phenol in the *A. dracunculus* hydrophilic extract was determined using the 10% Folin-Ciocalteu’s reagent (FCR). In a 100 mL volumetric flask, 7.5% sodium carbonate was prepared by adding 7.5 g of it and then filling the flask with distilled water up to volume. In the same way, a 0.1% solution of Gallic acid (standard control) was prepared by dissolving 100 mg in another 100 mL volumetric flask. Finally, different concentrations of the plant extract (100, 70, 50, 40, and 10 μg/mL) were prepared using the same previous method to make the reaction mixture. Then 2.5 mL of each primary reagent (sodium carbonate + Folin-Ciocalteu’s reagent) was added to 0.5 mL of plant extract in each tube, and the test tubes were incubated for 45 min at 45°C. Finally, the absorbance was read at wavelength 765 nm. Based on the measured absorbance, the concentration of the Gallic acid equivalent was determined to be in mg of GAEq/g for the working plant dry extract (Jaradat et al., 2015).

### 2.5 DPPH inhibitory assay

The *A. dracunculus* extract solution was diluted to maintain 100, 50, 20, 10, 5, and 2 μg/mL concentrations using methanol as solvent. Each test tube contained 1 mL of each concentration and was appropriately labeled. One ml of methanol and 1 ml of 0.002% methanolic DPPH solution were added to each test tube to prepare 3 mL as the final volume inside each test tube (caution: the preparation steps should be done with minimum light exposure because DPPH is light sensitive). The samples were incubated for 30 min in a dark place, and the spectrophotometer device determined their optical densities at a wavelength of 517 nm. The equation used in this analytical study to calculate the inhibition percentage is shown below:

$$\%\text{ DPPH inhibition}=\left(\text{AB}-\text{AE}\right)/\text{AB}\times 100\%$$

AB is the recorded absorbance of the blank solution; AE is the recorded absorbance of the *A. dracunculus* sample solution.

The plant extract’s DPPH free radical inhibitory half-maximal inhibitory concentration (IC_50_) was calculated utilizing BioDataFit edition 1.02 (Jaradat et al., 2016).

### 2.6 α-Amylase inhibition assay

This method was performed by utilizing the assay modified by McCue and Shetty (McCue and Shetty, 2004). The following dilutions were prepared: 10, 50, 70, 100, 500 μg/mL by dissolved plant extract in little milliliters of 10% DMSO and then another dissolved in buffer (0.02 M of Na_2_HPO_4_/NaH_2_PO_4_, 0.006 M NaCl, at pH 6.9) to give concentrations of 1,000 μg/mL. After that, two units/mL of porcine pancreatic α-amylase enzyme solution was prepared in 10% DMSO. A working solution was prepared by mixing 0.2 mL of enzyme solution with 0.2 mL of each *A. dracunculus* hydrophilic extract and was incubated for 10 min at 30°C. Following the incubation period, 0.2 mL of a freshly prepared 1% starch aqueous solution was added to each working solution. After that, the final solution was incubated for at least 3 min. A 0.2 mL dinitrosalicylic acid (DNSA), which is a yellow color reagent, was added to stop the reaction. The next step was diluting each working solution with 5 mL of distilled water and then boiling for 10 min in a water bath at 90°C. The mixture was then cooled to room temperature, and the absorbance was taken at 540 nm. The blank was prepared following the steps above, but the plant extract was replaced with 0.2 mL of the previously described buffer. Acarbose was used as the standard reference, following the same steps used for plant extract.

The following equation was used to calculate the α-amylase inhibitory activity

$$\%\;\alpha -\text{amylase inhibitory activity}=\left(\text{AB}-\text{AE}\right)/\text{ AB}*100\%$$

AB: is the absorbance of blank; AE: is the absorbance of *A. dracunculus*.

### 2.7 Porcine pancreatic lipase inhibition assay

This study used the porcine pancreatic lipase inhibitory method according to the method of (Bustanji et al., 2010). Five different solutions were prepared from a 500 μg/mL stock solution from the plant extract in 10% DMSO. The concentrations of these solutions were 50, 100, 200, 300, and 400 μg/mL respectively. Before use, a freshly stocked solution was prepared from 1 mg/mL of porcine pancreatic lipase enzyme in Tris–HCl buffer. *P*-nitrophenyl butyrate (PNPB) was prepared by dissolving 20.9 mg in 2 mL of acetonitrile. 0.1 mL of porcine pancreatic lipase and 0.2 mL of each diluted solution series for the plant extract were mixed. Then, a Tri-HCl solution was added to the resulting mixture to get a total volume of 1 mL. After that, the final mixture was incubated at 37°C for 15 min. The next step was the addition of 0.1 mL of PNPB solution to each test tube and incubated for 30 min at 37°C. The hydrolysis of the PNPB compound into p-nitrophenolate ions was measured at 410 nm using a UV spectrophotometer to determine the pancreatic lipase activity. Orlistat was used as a positive control compound by using the same procedure. The following equation was used in this analytical study.

$$\%\text{ lipase inhibition}=\left(\text{AB}-\text{AE}\right)/\text{AB}*100\%$$

Where AB is the recorded absorbance of the blank solution, AE is the recorded absorbance of the *A. dracunculus* sample solution.

### 2.8 Cytotoxicity MTS assay

In RPMI-1640 media enriched with 10% fetal bovine serum, 1% L-glutamine, and 1% Penicillin/Streptomycin antibiotics, cervical adenocarcinoma cells (HeLa) were cultured. The cells were grown in a 5% CO_2_ humidity at 37 C. The cells were seeded at 2.6 × 10^4^ cells/well in a 96-well plate. After 48 h, the cells were incubated with multiple concentrations of the plant sample (10, 5, 2.5, 1.25, and 0.625 mg/mL) for 24 h to be tested. Cell viability was assessed by Cell Tiltert 96^®^ Aqueous One Solution Cell Proliferation (MTS) Assay, depending on the manufacturer’s instructions (Promega Corporation, Madison, WI, United States). Shortly, at the end of the treatment, 20 μL of MTS solution per 100 μL of media was added to each well and incubated for 2 hours at 37 C. The absorbance was measured at 490 nm.

### 2.9 Statistical analysis

The mean values ± SD of standard deviations of all the findings of the *A. dracunculus* hydrophilic extract (DPPH free radical inhibitory, cytotoxic, anti-lipase, and anti-α-amylase activities) were calculated; results with a *p*-value of <0.05 were deemed significant. The unpaired *t*-test was used to analyze the data.

## 3 Results

### 3.1 Phytochemical screening

Secondary metabolic molecules are produced as bioactive precursors stored in plant tissues, which are released due to the plants’ reactions to environmental stressors (Bartwal et al., 2013). Therefore, we aimed to assess for species-based compounds and characteristics that could affect herbal medicines through the phytochemical screening methods for identifying and authenticating raw compounds. The phytochemical screening results revealed that the *A. dracunculus* hydrophilic extract contains only a phenolic group.

We next measured the total phenolic content using spectrophotometric techniques. A calibration curve of gallic acid was utilized to estimate the total phenol content in *A. dracunculus’s* aqueous extract. The following equation was used to compute the total phenol content from the dotted line obtained.

$$y=0.0117x+0.0195,R^{2}=0.9955$$

Where y is the absorbance at 765 nm, and x is the total phenol content of the plant extract.

The results revealed that the total phenol content of *A. dracunculus* aqueous extract is 0.15 ± 0.01 mg GAE/g of plant dry extract.

### 3.2 *A. dracunculus* DPPH free radical inhibitory activity

**FIGURE 2:**
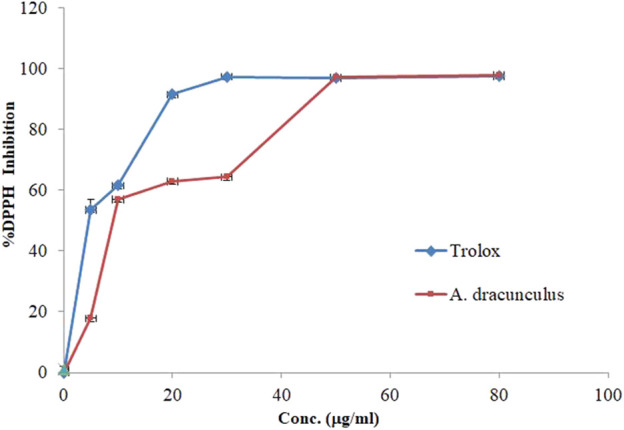
DPPH scavenging activity by Trolox and *A. dracunculus* hydrophilic extract.

The DPPH free radical inhibitory effect of *A. dracunculus* aqueous extract was estimated using DPPH assay. Figure 2 shows the hydrophilic plant extract with a linear positive dose-dependent free DPPH inhibitory effect. However, the results showed that *A. dracunculus* aqueous extract has a potent DPPH free radical inhibitory effect with an IC_50_ dose of 10.71 ± 0.01 μg/mL compared with the potent DPPH free radical inhibitory drug Trolox, which has a DPPH free radical inhibitory IC_50_ value of 5.7 ± 0.92 μg/mL.

### 3.3 α-Amylase inhibition assay showed limited activity in the *A. dracunculus* extracts

An *in vitro* assay of α-amylase inhibitory was conducted on *A. dracunculus* hydrophilic extract activity using Acarbose as a positive control and starch as a substrate. The results of α-amylase inhibitory activity for the screened plant and Acarbose illustrated that the *A. dracunculus* hydrophilic extract has very weak α-amylase inhibitory activity with IC_50_ value > 1,000 μg/mL compared with commercial antidiabetic drug Acarbose (IC_50_ = 28.18 ± 1.27 μg/mL).

### 3.4 Porcine pancreatic lipase inhibitory property of *A. dracunculus*

**FIGURE 3:**
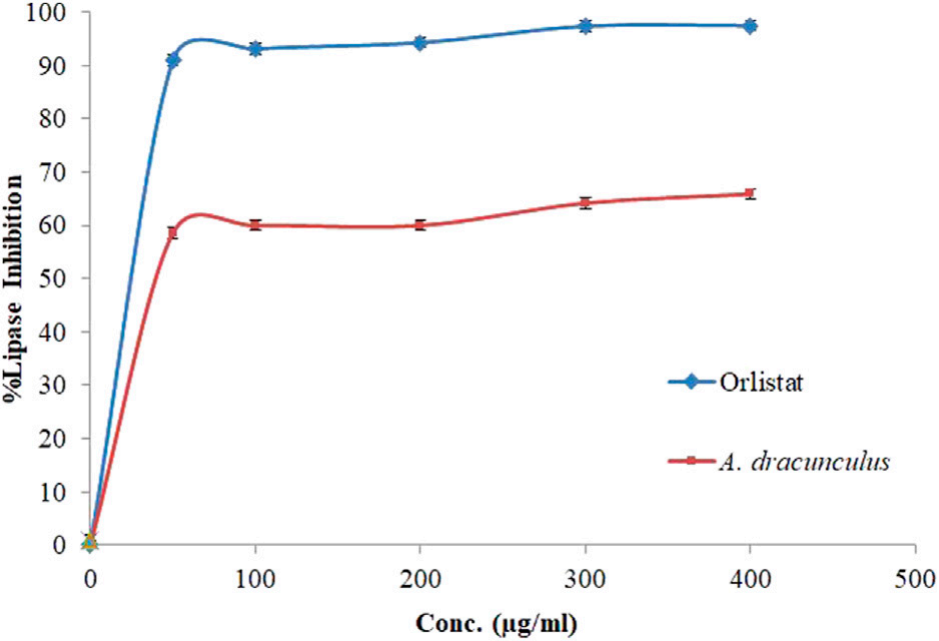
Porcine pancreatic lipase inhibition activity of A. dracunculus hydrophilic extract and Orlistat samples.

The hydrolysis of *p*-nitrophenyl butyrate to *p*-nitrophenol was used to measure the influence of *A. dracunculus* hydrophilic extract on the porcine pancreatic lipase enzyme. The assay worked by comparing it with a potent lipase inhibitory agent, Orlistat. The lipase enzyme inhibitory activity of *A. dracunculus* aqueous extract and Orlistat also results in their IC_50_ values, which are shown in Figure 3. The results showed that *A. dracunculus* and Orlistat samples inhibited pancreatic lipase in a concentration-dependent manner, with IC_50_ values of 60.25 ± 0.33 and 12.3 ± 0.35 μg/mL, respectively (*p*-value < 0.05). This means that *A. dracunculus* aqueous extract has a potential antilipase effect.

### 3.5 Cytotoxic effects of *A. dracunculus*

**FIGURE 4:**
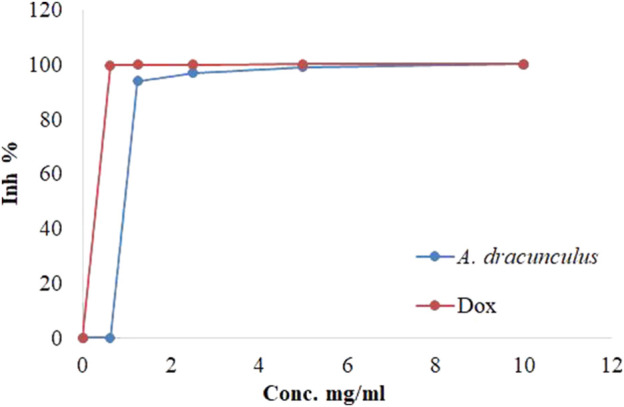
Inhibitions percentages of HeLa cancer cells by *A. dracunculus* aqueous extract and Doxorubicin.

We next sought to assess the potential anticancer effects of the *A. dracunculus* through the use of HeLa cancer cells (cervical carcinoma line). The treatment results of HeLa cancer cells with five concentrations of *A. dracunculus* aqueous extract. Our results showed that this extract has potential cytotoxic activity against the HeLa cancer cell line compared with the potential chemotherapeutic agent Doxorubicin, as illustrated in Table 1; Figure 4. However, the results showed that the obtained IC_50_ doses were 1.032 ± 0.33 and 0.625 ± 0.03 mg/mL, respectively. However, at a 10 mg/mL concentration, the *A. dracunculus* aqueous extract has the same percent of inhibition as Doxorubicin (99.9%).

**TABLE 1:** Percents of inhibitions and IC_50_ values (mg/mL) of *A. dracunculus* aqueous extract and Doxorubicin against HeLa cancer cells.

Concentrations	*A. dracunculus*	Doxorubicin
10	99.9 ± 0.97	99.9 ± 0.13
5	98.87 ± 0.19	99.9 ± 0.53
2.5	96.88 ± 0.77	99.8 ± 1.1
1.25	93.73 ± 0.81	99.7 ± 0.28
0.63	0.01 ± 0.03	99.6 ± 0.92
0	0 ± 0.00	0 ± 0.00
IC_50_	1.032 ± 0.33	0.625 ± 0.03

## 4 Discussion

Many researchers are working to discover new sources of naturally occurring bioactive chemicals or their derivatives (Ukrainets et al., 2006). Phenolic compounds are particularly noteworthy because of their anticarcinogenic, anti-obesity, antioxidative, antibacterial, and fungal properties. By their widespread distribution and specific properties, natural phenolic compounds have emerged as essential factors in research into biological and pharmaceutical applications (Tanase et al., 2019). According to the findings of this study, the preliminary phytochemical screening revealed the presence of phenolic compounds, while the total phenol content assay showed that *A. dracunculus* hydrophilic extract contains 0.15 ± 0.01 mg GAE/g of plant dry extract.

A study by Behbahani *et al.* found that the total phenolic content of *A. dracunculus* aqueous-ethanolic extract equaled 24.10 mg GAE/g (Behbahani et al., 2017). Another study by Rajabian *et al.* showed that *A. dracunculus* aqueous extract’s total phenol content was 9.30 ± 0.11 mg tannic acid equivalent/g dry aerial parts (Rajabian et al., 2017). Compared with these studies, our investigated *A. dracunculus* aqueous extract has smaller amounts than these, which may refer to geographical location and other factors affecting the secondary metabolites’ contents.

The DPPH free radical scavenging test is a commonly used method to assess the antioxidant properties of substances by measuring their capacity to scavenge free radicals or donate hydrogen. It is known for being quick, simple, cost-effective, and frequently utilized in evaluating the antioxidant activity of various substances (Munteanu and Apetrei, 2021). The DPPH free radical scavenging assay results of *A. dracunculus* hydrophilic extract showed that it has a potent DPPH free radical inhibitory effect with an IC_50_ dose of 10.71 ± 0.01 μg/mL compared with the potent DPPH free radical inhibitory drug Trolox, which has a DPPH free radical inhibitory IC_50_ value of 5.7 ± 0.92 μg/ml. A study performed by Mumivand *et al.* found that the total phenol content from *A. dracunculus* aqueous methanolic extract from Iran (Isfahan) was 68.54 ± 3.5 µg GAE/g^-1^ of plant extract and has anti-DPPH activity with an IC_50_ value of 0.069 ± 0.014 mg/mL^-1^ (Mumivand et al., 2017).

In fact, the mechanism of action of phenolic compounds as DPPH free radical inhibitors involves their ability to donate electrons and neutralize the highly reactive DPPH radical. Phenols contain multiple electron-rich phenolic hydroxyl groups that can readily donate electrons to stabilize free radicals. When polyphenolic compounds encounter DPPH radicals, they undergo a redox reaction wherein they transfer electrons to the DPPH molecule, converting it into a stable, non-radical form. This electron donation interrupts the free radical formation chain reaction and neutralizes the oxidative stress caused by DPPH radicals. The specific structural features of polyphenols, such as the number and arrangement of phenolic rings and the presence of conjugated double bonds, influence their efficacy in scavenging DPPH radicals. Additionally, the hydrogen-donating capacity of hydroxyl groups plays a crucial role in the overall antioxidant activity of polyphenolic compounds. The complex and varied structures of phenols contribute to their versatility in combating oxidative stress, making them valuable agents in preventing free radical-induced cellular damage (Kumarappan et al., 2012).

At the same time, *A. dracunculus* aqueous extract showed a weak anti-α-amylase effect. An investigation carried out by Güvenalp et al. concluded that aerial parts of *A. dracunculus* methanol extract have an α-amylase inhibitory activity with an IC_50_ dose of 5.45 ± 0.06 μg/mL (Güvenalp et al., 2017).

To the best of our knowledge, no investigations have been conducted to evaluate the antilipase effect of the *A. dracunculus* plant, and our study is the first one that deals with this experimental work.

Our results suggest that *A. dracunculus* aqueous extract possesses a concentration-dependent antilipase effect, as demonstrated by its IC_50_ value (60.25 ± 0.33 μg/mL) compared with Orlistat (IC_50_ = 12.3 ± 0.35 μg/mL) which indicates that this extract has the potentials as a valuable natural resource in the context of lipid metabolism modulation.

As stated in several studies, phenolic molecules demonstrated the ability to interfere with pancreatic lipase activity. They can effectively inhibit lipid digestion and absorption by forming complexes with the enzyme or modulating its activity. This inhibition decreases the availability of free fatty acids for absorption, contributing to regulating body weight and preventing obesity-related complications (Buchholz and Melzig, 2015).

In our data, *A. dracunculus* hydrophilic extract showed a cytotoxic effect against HeLa cancer cells compared with Doxorubicin. A study conducted by Hong and Ying showed that *A. dracunculus* possesses potent anticancer effects by inducing DNA damage in these cells (Hong and Ying, 2015). Moreover, Tüylü et al. concluded that *A. dracunculus* essential oil has substantial cytotoxic activity in human lymphocytes (Tueylue et al., 2009).

The cytotoxic effects of phenolic compounds are primarily attributed to their ability to induce apoptosis, inhibit cell proliferation, and disrupt key cellular processes in cancer cells. Additionally, phenolic compounds demonstrate antioxidant properties, contributing to their potential to prevent oxidative stress-related cellular damage that could lead to cancer development (Abotaleb et al., 2020).

In fact, the presence of phenolic compounds in the *A. dracunculus* hydrophilic extract provides the plant with potential biological capacities as well as these molecules have been considered more powerful antioxidative, antilipase, and cytotoxic agents than some commercial synthetic drugs (Dai and Mumper, 2010; Gulua et al., 2018).

It's important to note that the effectiveness might not be attributed to a single compound but could involve multiple phenolic compounds working together synergistically. This synergy can enhance the overall pharmacological impact, making the combined effect more potent than the sum of the individual effects of each compound.

Our results could be used to design and develop remedies for oxidative stress, obesity, and cancer diseases. The constraints inherent in our study pertain to the requisite clinical and preclinical examinations necessary to substantiate our research outcomes and to elucidate the pharmacokinetic and dynamic efficacy of *A. dracunculus* hydrophilic extract. Furthermore, instrumental analysis, including LC-MS-MS, is imperative for identifying the specific bioactive constituents of *A. dracunculus* hydrophilic extract that underlies its antilipase, cytotoxic, and free radical scavenging properties.

## 5 Conclusion

Overall, the current study explored the phytochemical constituents, DPPH free radical inhibitory, anti-α-amylase, cytotoxic properties, and, for the first time, the anti-lipase effects of *A. dracunculus* hydrophilic extract. The results revealed that the phenolic group is the only phytochemical constituent, and the total phenolic contents were 0.15 ± 0.01 mg GAE/g of plant dry extract. Compared with positive controls, *A. dracunculus* extract has potent DPPH free radical inhibitory, antilipase, and cytotoxic effects but weak anti-α-amylase effects. These results disclose the possible application of *A. dracunculus* hydrophilic extract to treat oxidative stress, cancer, and obesity. However, its role in the management of diabetes mellitus is less evident. Additional, in-depth phytochemical analyses and *in vivo* pharmacological studies are urgently needed to explore the importance of *A. dracunculus* as a medicinal plant.

## Data Availability

The raw data supporting the conclusion of this article will be made available by the authors, without undue reservation.
